# Supplementary material for: The Relationship between Endophytic Fungi of Chimonanthus praecox and Volatile Metabolites under Different Circadian Rhythms and Blooming Stages
Source: J Fungi (Basel). 2024 Feb 11;10(2):145. doi: 10.3390/jof10020145 (PMC10890430; doi:10.3390/jof10020145)
Supplement: Supplementary file 1 [file jof-10-00145-s001.zip › jof-2838528-supplementary.pdf]

Supplementary marterials

Relationship between Endophytic Fungi of *Chimonanthus praecox* and Volatile Metabolites Under Different Circadian Rhythms and Blooming Stages

Yue Li [1], Jingying Hei [2], Xiahong He [2], Rui Rui [1,2*] and Shu Wang [1,2*]

Table S1. Summary of sample sequencing information

Sample	Sequence Number	Base Number	Average Length	Shortest Sequence Length	Maximum sequence Length
GUA	72546	17717762	376	360	465
GUB	74339	17883713	41	158	468
GUC	71521	17182720	240	164	448
GOA	73417	17209111	234	153	501
GOB	71944	16810275	234	145	512
GOC	72771	17515452	241	171	453

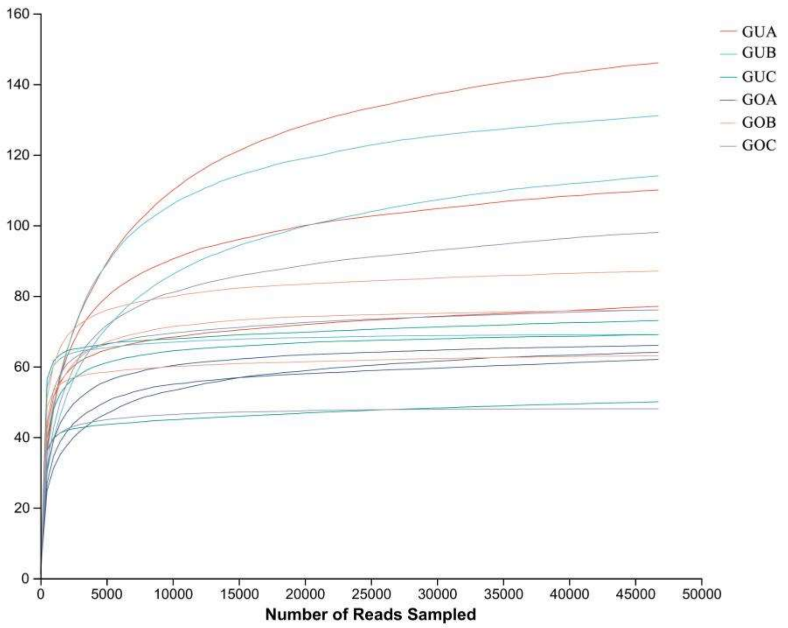

Figure S1. Sequencing data results in endophytic fungi of *C. praecox* OTU dilution curve

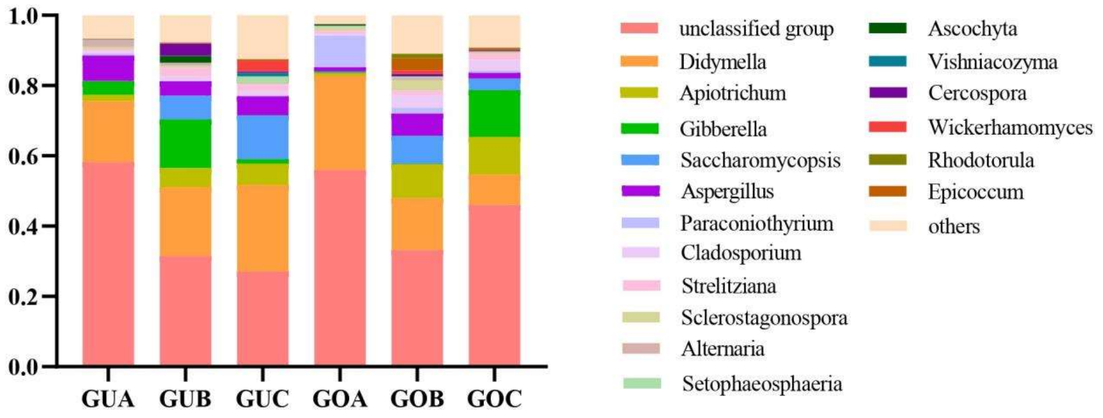

Figure S2. Community structure of endophytic fungi of *C. praecox* at genus level.

Table S2. Floral volatile emitted from *C. praecox* with different blooming stages and circadian rhythms

*RT**(min.)	*LRI**	Compounds	7:00 AM	13:00 PM	19:00 PM
3.92	865	Cyclohepta-1,3,5-triene	1.13/0.33	0.69/0.33	0.79/0.3
12.36	868	α-Terpinene	-/1.72	0.11/-	-/-
46.18/40.16	951	Germacrene D	0.05/0.13	1.62/-	0.01/0.47
27.24	839	Benzyl alcohol	0.71/20.42	1.51/28.62	-/24.05
21.85	893	(-)-Myrtenol	5.74/30.49	23.15/41.78	-/31.29
7.07	868	Ethylbenzene	0.45/0.07	0.19/0.18	2.19/0.66
7.05	875	M-Xylene	2.08/-	-/-	-/-
7.10	861	P-Xylene	-/-	1/-	-/-
42.97	911	Eugenol	-/3.21	-/4.2	-/3.95
9.81/14.23	882	O-toluic acid	1.42/0.63	-/0.04	-/Tr

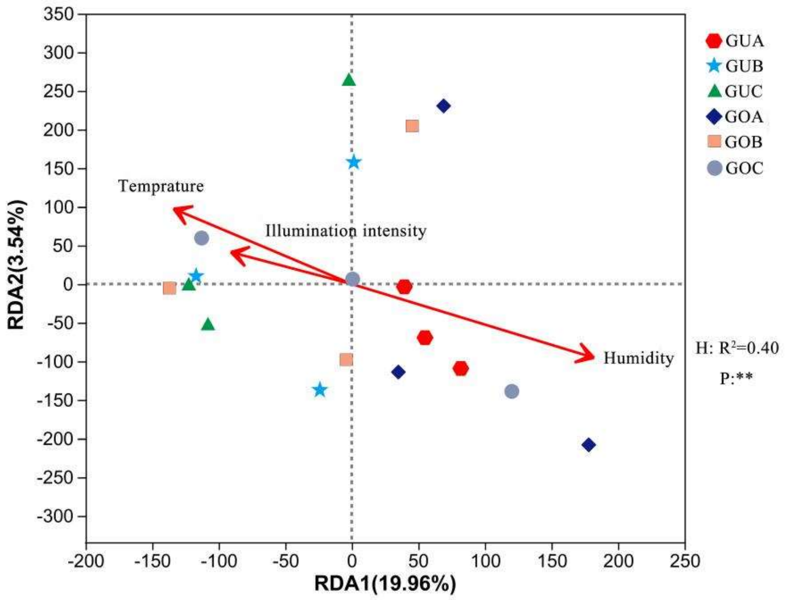

Figure S3. Correlations between the endophytic fungal community in *C. praecox* and environmental factors (T: temperature; H: humidity).

Table S3. Correlations between endophytic fungal diversity in *C. praecox* with environmental factors.

	GU	GO	Total
Temperature	0.543	0.556	0.550
Humidity	0.882*	0.883*	0.882*
Illumination intensity	0.715	0.715	0.715
